# Carcinosarcoma: A Rare and Aggressive Breast Tumor in a Young Lady

**DOI:** 10.7759/cureus.37003

**Published:** 2023-04-01

**Authors:** Poh Nuan Leng, Wan Zainira Wan Zain, Mohd Azem Fathi Mohammad Azmi, Maya Mazuwin Yahya, Khairul Anuar Azis, Siti Fatimah Noor Mat Johar, Wan Azman Wan Sulaiman, Dumin Balingi, Sharifah Emilia Tuan Sharif, Wan Faiziah Wan Abdul Rahman, Lau Chiew Chea, Juhara Haron

**Affiliations:** 1 Department of Surgery, School of Medical Sciences, Universiti Sains Malaysia, Kota Bharu, MYS; 2 Breast Cancer Awareness & Research Unit, Hospital Universiti Sains Malaysia, Kota Bharu, MYS; 3 Department of Plastic and Reconstructive Surgery, School of Medical Sciences, Universiti Sains Malaysia, Kota Bharu, MYS; 4 Department of Pathology, School of Medical Sciences, Universiti Sains Malaysia, Kota Bharu, MYS; 5 Department of Radiology, School of Medical Sciences, Universiti Sains Malaysia, Kota Bharu, MYS

**Keywords:** breast cancer biology, breast cancer, mastectomy, metaplastic breast carcinoma, carcinosarcoma of the breast

## Abstract

Carcinosarcoma of the breast is a subtype of metaplastic breast carcinoma characterized by differentiation of the neoplastic epithelium toward mesenchymal-looking elements. It is a highly aggressive rare subtype of invasive breast neoplasm that exhibits a distinct histologic entity. Only a limited number of reports related to this type of disease have been reported. Here, we present a case of breast carcinosarcoma in a lady in her early 20s, which is relatively young among all cases published. It was challenging to achieve diagnosis preoperatively with histopathological evaluation of the ultrasound-guided tru-cut biopsy sample. With no evidence of distant metastasis clinically and radiologically, a surgical option was opted for. Left mastectomy and left chest wall reconstruction with deep inferior epigastric artery free flap were performed. Post-excision specimen was confirmed to be carcinosarcoma.

## Introduction

Carcinosarcoma of the breast often manifests as a rare and highly aggressive type of tumor with a prevalence of <1% of all newly diagnosed breast cases every year. Metaplastic carcinoma may display monophasic or biphasic morphology but carcinosarcoma refers to the neoplastic epithelium of invasive carcinoma subtly merged with a highly cellular, mitotically active pleomorphic spindle cell sarcoma. Usually, the demarcation between the carcinomatous and sarcomatous components is unclear under light microscopic fields without a definite transition zone between these two types of malignant cells [1]. To date, limited data are available regarding the treatment modalities [2]. This category of breast tumors presents with histology of a mixture of adenocarcinoma with spindle cell, squamous, chondroid, or bone-forming neoplastic cells. Mostly, it is negative for estrogen receptor (ER), progesterone receptor (PR), and human epidermal growth factor 2 (HER2-neu) receptor, at the same time, it favors a worse prognosis than triple-negative breast malignancies with fewer treatment choices [3]. Here, we are reporting a case of carcinosarcoma of the left breast in a 24-year-old young lady who presented with a rapid, aggressive course of the disease. Achieving the diagnosis is challenging initially due to its clinical features and difficulty in obtaining the histopathological confirmation preoperatively.

## Case presentation

**Figure 1:**
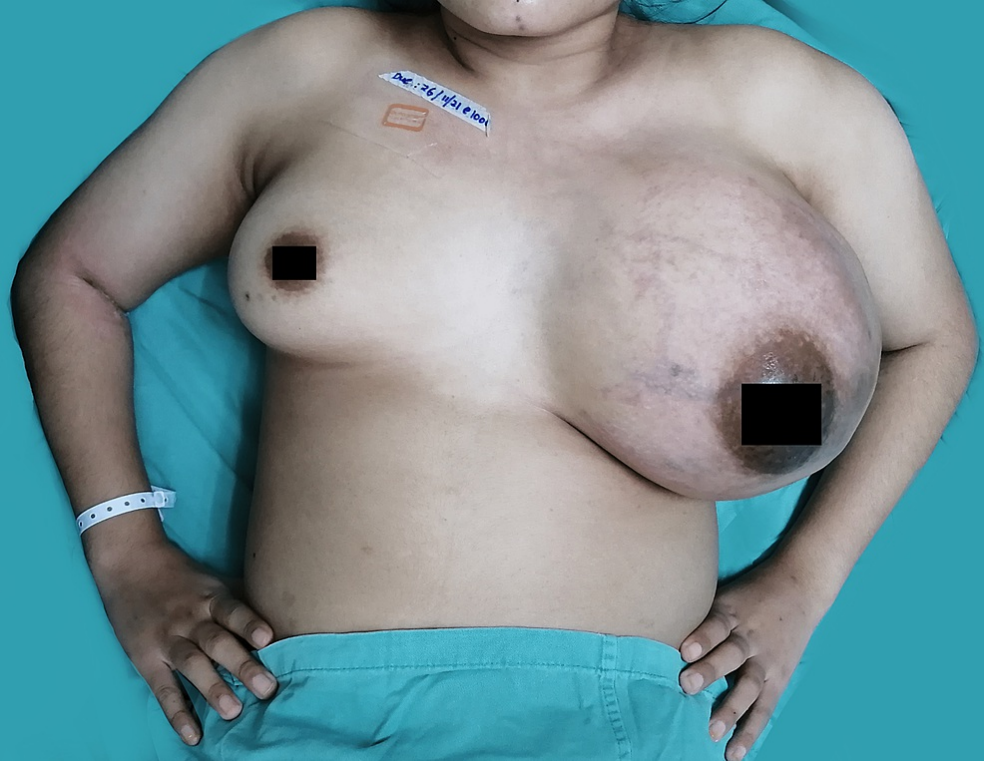
Patient’s first presentation. Her left breast measures 20 × 20 cm, with shiny and tense skin and dilated veins. A fentanyl patch of 25 µg is attached at the right infraclavicular region for analgesic control.

A 24-year-old single, nulliparous woman with no known risk factors presented with a lump in the left breast of six months duration. The mobile lump was 1-2 cm in size and became painful during menstruation. It increased rapidly in one month and caused discomfort. Physical examination revealed a tense, hard, fixed 20 × 20 cm left breast swelling with shiny skin and dilated veins. Neither ulceration nor nipple discharge was seen (Figure 1). There was no palpable lymph node and no apparent distant metastasis clinically. Blood investigations showed anemia of chronic disease concomitant with iron deficiency anemia and thrombocytosis, as well as coagulopathy and hypoalbuminemia. Lactate dehydrogenase was raised.

**Figure 2:**
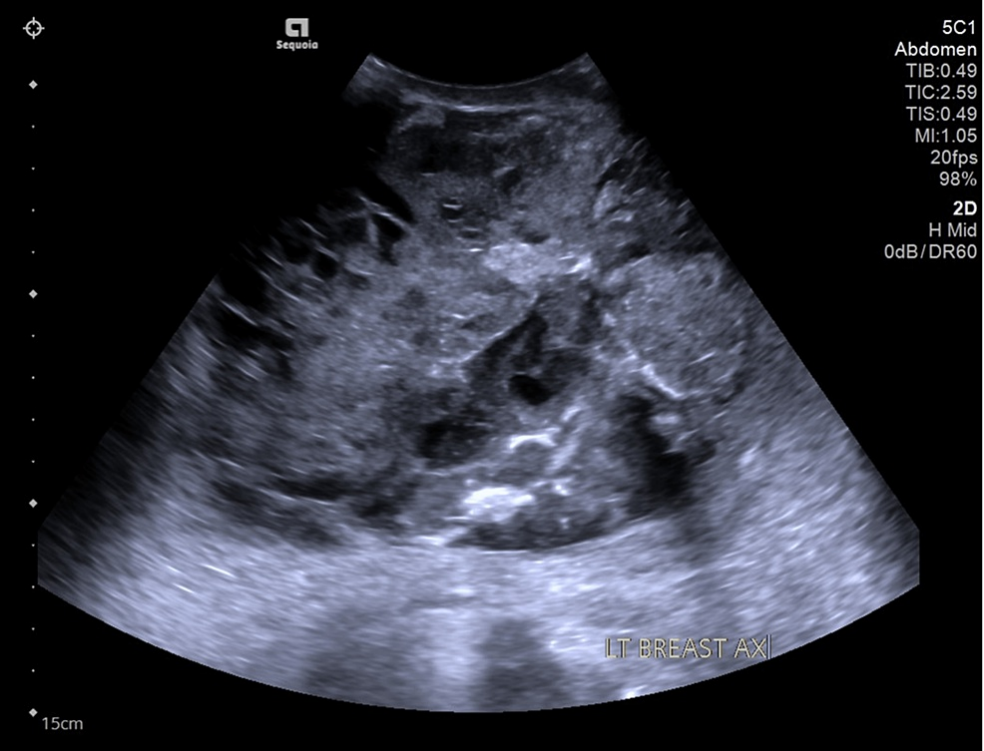
Ultrasound of the left breast showing a large heterogeneous solid mass with cystic components within.

Ultrasonography of the left breast revealed a well-defined, heterogeneous, solid cystic mass measuring 10.6 cm × 14.1 cm × 15.3 cm (AP × W × CC) in which the cystic area represented the necrotic center or hemorrhagic component (Figure 2). The characteristics and size of the mass were interpreted as Breast Imaging-Reporting and Data System (BI-RADS) 4C which signified high suspicion of malignancy and warranted tissue diagnosis. An ultrasound-guided tru-cut biopsy of the mass returned as a malignant tumor with mesenchymal differentiation, with differential diagnoses including malignant phyllodes or metaplastic carcinoma.

**Figure 3:**
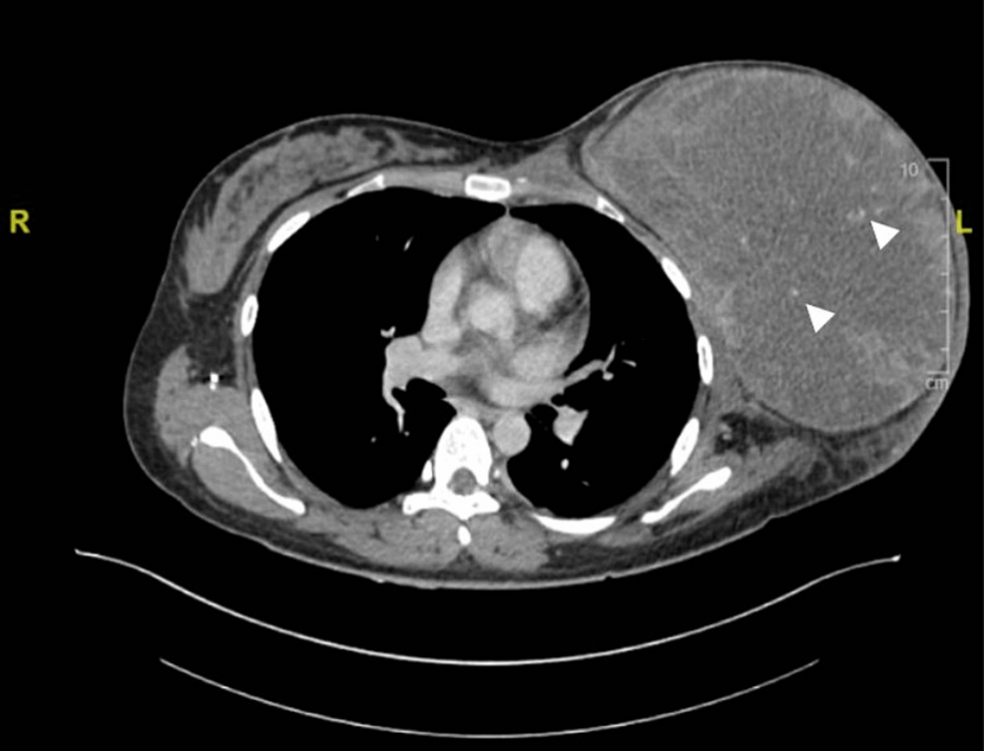
Contrast-enhanced computed tomography of the thorax showed a large, well-defined, heterogeneously enhancing left breast mass with central non-enhancement suggestive of necrosis and foci of calcification within (arrowheads).

Preoperative contrast-enhanced computed tomography (CT) of the thorax, abdomen, and pelvis was performed. The CT showed a large, well-defined, heterogeneously enhancing left breast mass with central non-enhancement suggestive of necrosis and foci of calcification within (Figure 3). The mass measured 12.9 cm × 19.4 cm × 18.8 cm (AP × W × CC). Features of local involvement such as skin thickening and a poor plane of demarcation with underlying pectoralis muscle were seen. No distant metastasis was observed on the CT.

**Figure 4:**
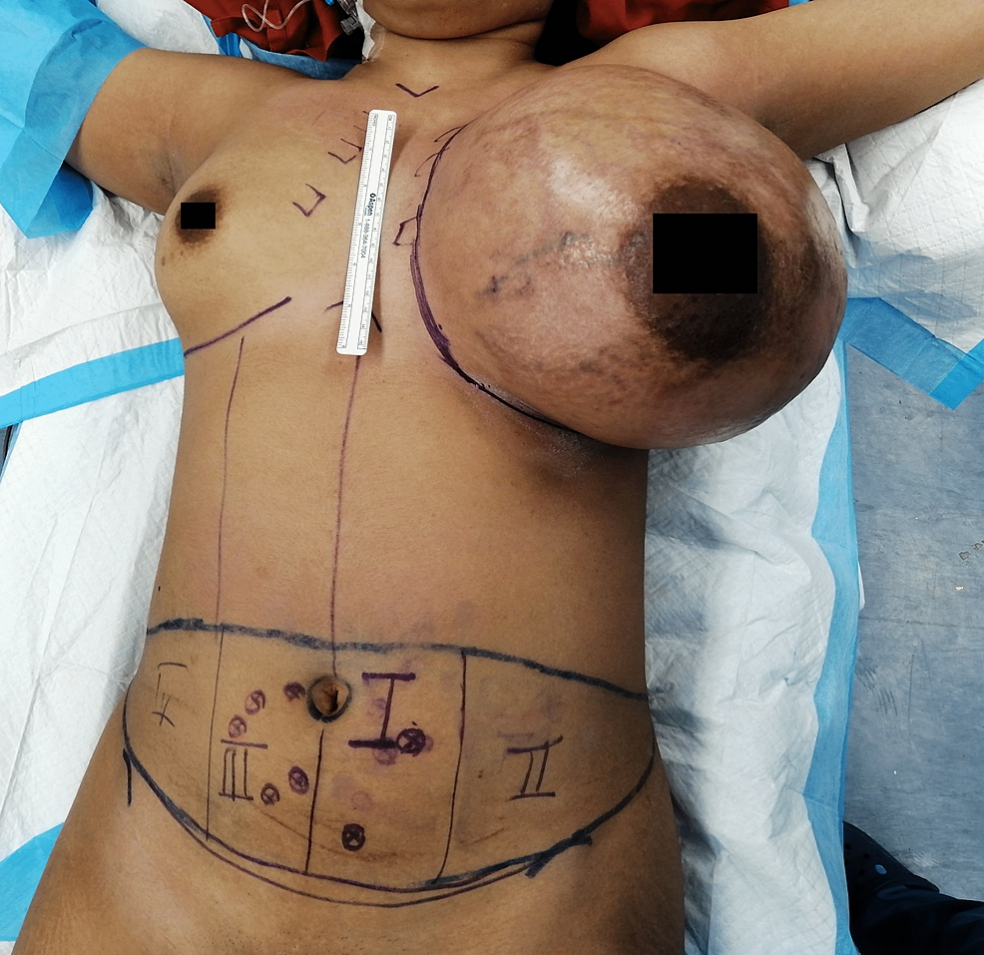
Preoperative image in the operation theater after surface markings had been done.

**Figure 5:**
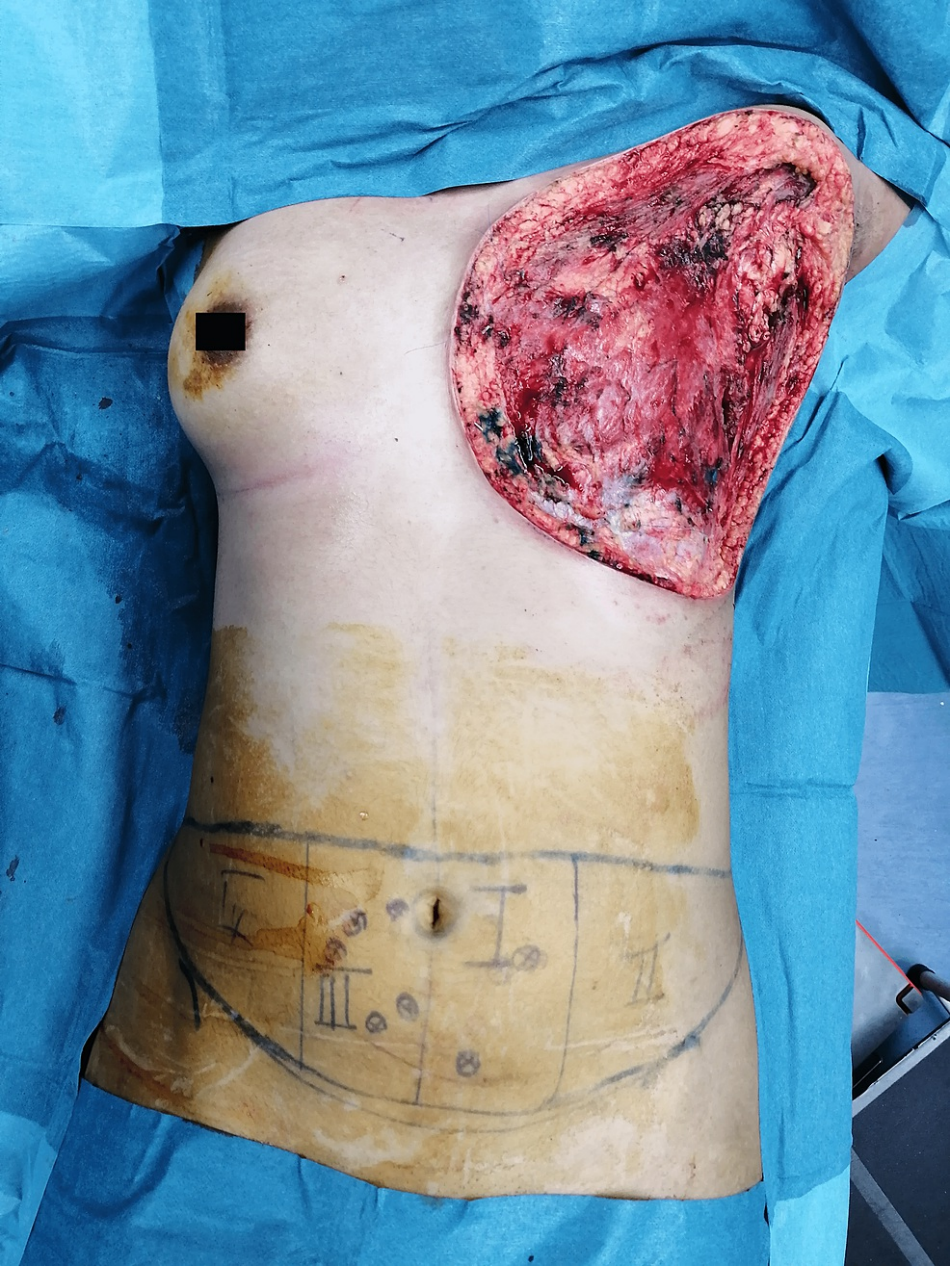
Intraoperative image after left mastectomy, right before left chest wall reconstruction with deep inferior epigastric artery free flap was performed.

The left breast mass rapidly grew over two weeks. With evidence of probable mesenchymal tumor, a left mastectomy and left chest wall reconstruction with deep inferior epigastric artery free flap were performed. Intraoperatively, the 25 × 25 cm left breast weighed 4.8 kg with both pectoralis major and minor muscles, and a part of the latissimus dorsi was removed (Figures 4, 5). Due to the massive tumor size, maximum removal was attempted as much as possible. The wound had no skin flap as usual mastectomy wound, and it extended medially near the medial border of the sternum, superiorly near the left clavicle, laterally near the posterior axillary line, and inferiorly far from the inferior mammary line (Figure 5). Macroscopically, the margins of excision were 30 mm from the superior margin, 20 mm from the inferior margin, 50 mm from the lateral margin, 50 mm from the medial margin, and 10 mm from the deep margin. Soft tissue was reconstructed using a deep inferior epigastric artery perforator (DIEP) flap. Intraoperatively, the internal mammary vein was small in caliber, thus anastomosis was done with the pectoral branch of the thoracoacromial artery and venae commitantes. Zone I-III was incorporated in the pedicle, and zone IV was discarded before the flap inset. A specimen of the left breast was sent for histopathological examination (HPE).

**Figure 6:**
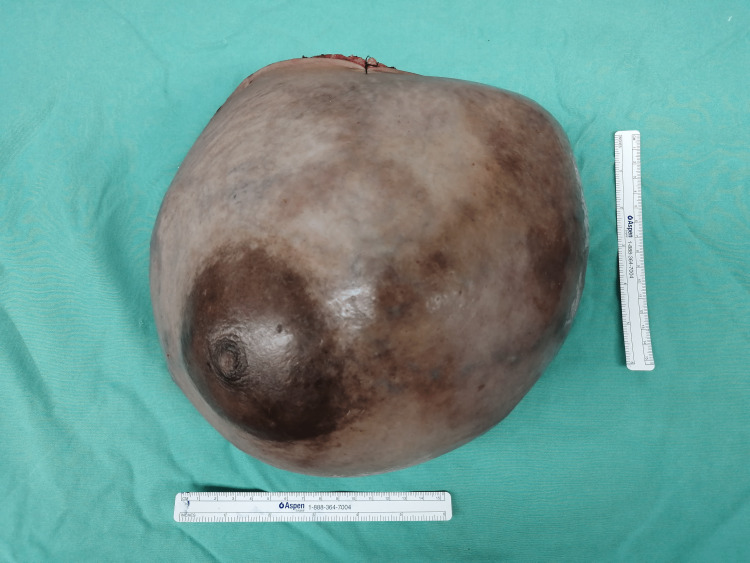
Gross specimen of left mastectomy measuring approximately 25 × 25 cm and weighing 4.8 kg was sent for histopathological examination.

**Figure 7:**
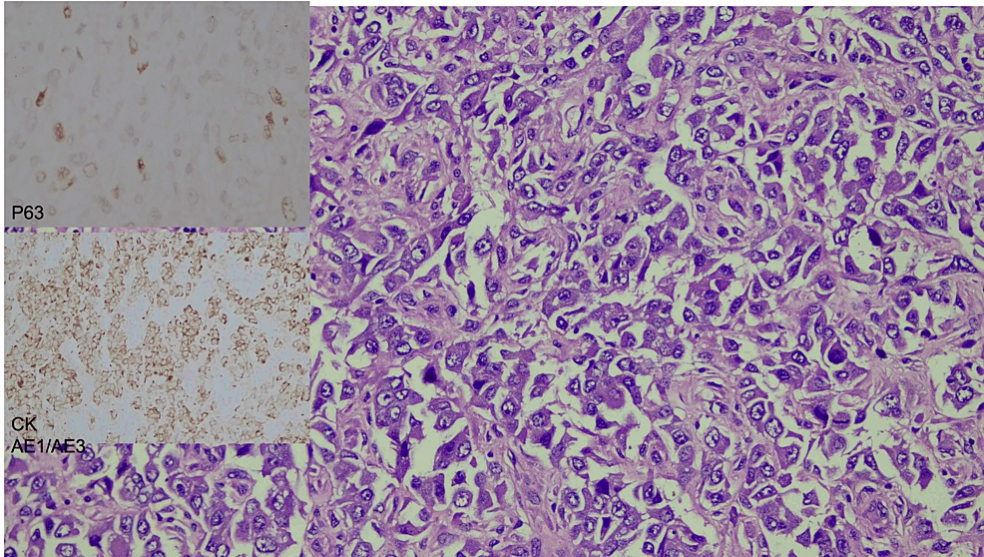
Histopathological examination. The malignant epithelial component was arranged in clusters, cords, and vague glandular patterns highlighted by positive CKAE1/AE3 immunohistochemical staining with focal P63 stain. The epithelial cells displayed marked pleomorphism, having vesicular nuclei, prominent nucleoli, and eosinophilic ample cytoplasm with frequent mitosis (hematoxylin and eosin, CKAE1/AE3, and P63 stains, ×200 magnification).

**Figure 8:**
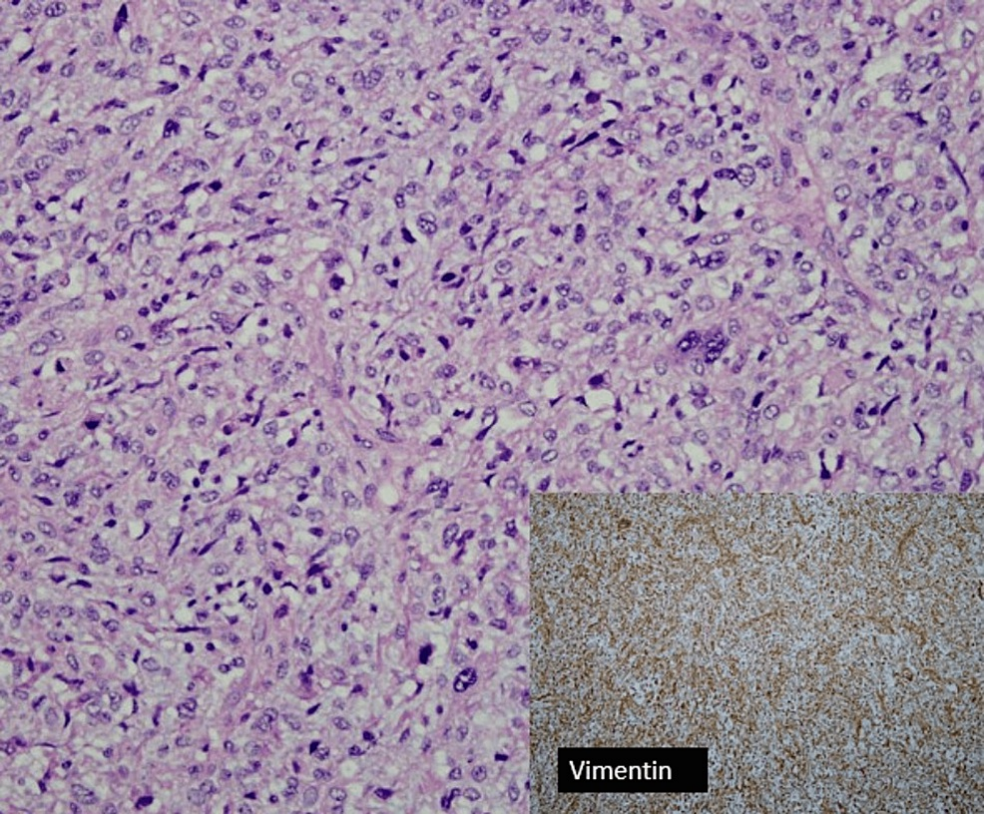
Histopathological examination. Malignant mesenchymal component arranged in diffuse sheets with moderate-to-marked pleomorphism (hematoxylin and eosin stain, ×200 magnification). This sarcomatous area is strongly positive for vimentin (bottom right insert). Mitosis is easily seen.

Grossly, a huge breast sample with an inverted nipple was sent (Figure 6). Upon sectioning, there was a well-circumscribed tumor occupying the whole breast measuring 26 × 18 × 17 cm. The tumor displayed a tan-brownish cut surface with an area of hemorrhage and necrosis. HPE of the tumor showed malignant epithelial and mesenchymal components with extensive areas of necrosis. The epithelial component was arranged in trabeculae, cord, and singly dispersed pattern highlighted by CKAE1/AE3 immunohistochemical stain with focal P63 stain (Figure 7). The cells displayed marked pleomorphism, having vesicular nuclei, prominent nucleoli, and eosinophilic cytoplasm with frequent mitosis. The mesenchymal component was arranged in diffuse sheets, highlighted by a vimentin stain (Figure 8). In some areas, there was an admixture of heterologous components of rhabdomyoblastic, osseous, and lipoblast differentiation. Both components were negative for ER, PR, HER2, cytokeratin (CK) 5/6, chromogranin, and synaptophysin.

**Figure 9:**
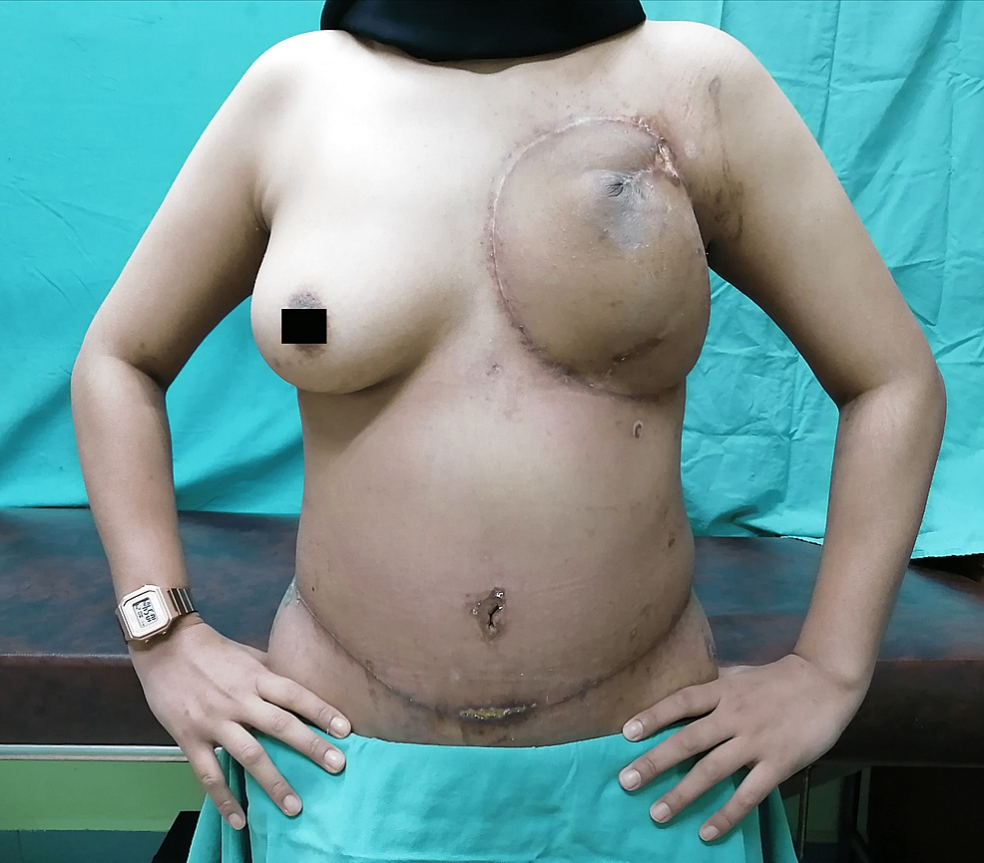
Operative sites seven weeks after the surgery.

Postoperatively, she was complicated with hospital-acquired pneumonia and DIEP flap partial necrosis. She responded well to antibiotics and minor debridement over the DIEP flap. Following the recovery, primary closure of the flap was done. She was seen in both surgery and plastic surgery outpatient clinics. As she had undergone chest wall reconstruction and had a small necrotic area postoperatively, her follow-up was scheduled more frequently on a twice-weekly basis until her wound was fully healed. She was planned for a three-monthly follow-up in the clinic, with an ultrasound of the contralateral breast, as well as six-monthly and yearly CT scans. On her follow-up seven weeks post-operation, she had a satisfactory recovery over the operation sites and she was delighted (Figure 9). Chemotherapy was offered; however, she refused initially and only agreed after four months of surgery. She was planned for three cycles of fluorouracil, epirubicin, and cyclophosphamide (FEC) regime and three cycles of taxane’s regime followed by radiotherapy 40 Gy/15#. Unfortunately, she received only one cycle of the FEC regime and defaulted on follow-up after the first chemotherapy. Therefore, the total duration of follow-up for the patient was only nine months after the surgery and she did not attend our clinic appointments after that.

## Discussion

Carcinosarcoma of the breast is a rare and exceptionally aggressive subtype of metaplastic breast cancer. Statistically, it has a 0.1-0.2% incidence rate of all malignant breast cancers [3]. According to the 2011 World Health Organization (WHO) Working Group, squamous cell carcinoma, spindle cell carcinoma, low-grade adenosquamous carcinoma, fibromatosis-like metaplastic carcinoma, and carcinosarcoma are the five distinct subtypes of metaplastic breast cancer [1]. However, the latest edition of the WHO classification of breast tumors does not recommend this terminology and classifies metaplastic carcinoma into six subtypes, namely, low-grade adenosquamous carcinona, fibromatosis-like metaplastic carcinoma, spindle cell carcinoma, squamous cell carcinoma, metaplastic carcinoma with heterologous mesenchymal differentiation, and mixed metaplastic carcinomas. Metaplastic carcinoma displays a variety of tumor differentiations into squamous or mesenchymal-looking elements, ranging from spindle, chondroid, osseous, and rhabdomyoid cells which can mingle with the usual carcinoma type [4]. The majority of the cases are negative for ER, PR, and HER2-neu receptor overexpression and are even more intrusive compared to triple-negative breast cancers [5].

According to the literature, the age at diagnosis ranges from 32 to 77 years old [6]. Our patient was 24 years old, which is the youngest reported case yet. As in most breast neoplasm cases, the most frequent complaint which prompted the patient to seek medical attention was a painful breast lump. In our case, the patient initially disregarded the left breast lump as it did not cause significant disturbance to her daily life. Later, the tumor started to show its malignant characteristics and the patient began to experience intolerable pain from the fast-growing breast lump. For this reason, patients tend to visit late when the disease is advanced.

In breast carcinosarcoma, breast imaging is not sufficient to achieve a diagnosis of the tumor. Due to the poorly differentiated characteristic of the tumor, reaching a preoperative diagnosis of carcinosarcoma is challenging if based solely on the histopathological interpretation of pre-surgery biopsy specimens. In addition, the diagnosis from the frozen section has a minimal role in the case of breast carcinosarcoma as the small selected piece of tissue is unable to signify the entire profile of the tumor [6]. Therefore, in our case, the final diagnosis was confirmed with the whole excised specimen post-mastectomy. Moreover, it is not uncommon for carcinosarcoma to be diagnosed after definitive surgery.

As a consequence of the tumor being malignant, the large tumor size often causes skin or chest wall fixation, nipple retraction, and ulceration [2]. In our case, the tumor was rapidly enlarging and stretching the surrounding structures, as well as invading the pectoralis muscles. Left mastectomy and left chest wall reconstruction with DIEP flap were performed on this young small built lady. Five margins need to be marked for specimen management according to Malaysian Clinical Guidelines 2019 in breast surgeries, namely, superior, inferior, lateral, medial, and deep margins. Meanwhile, due to the large wound, the latissimus dorsi myocutaneous flap could not cover the defect. Instead, the DIEP free flap was a preferable option to reconstruct the breast to give her a better body image, and, as a result, the patient was satisfied with the cosmetic results.

For the outcomes of the disease, important prognostic factors including tumor size more than 5 cm, lymph node involvement, and high Ki-67 >14% were significantly related to five-year overall survival, indicating a poor prognosis. In more advanced stages, carcinosarcoma of the breast is prone to have more percentage of local recurrence and distant metastasis, specifically in the setting of squamous cell carcinoma in lymph node metastasis, involvement of skin, and younger age group [7]. Generally, the cumulative five-year survival rate for this type of tumor is 49%, as reported by Wargotz et al. [8].

Identifying malignant epithelial and non-glandular components is required for histological diagnosis of metaplastic carcinoma [9]. These include squamous differentiation and/or differentiation into mesenchymal elements. The two components can both be metaplastic histology, or there can be one metaplastic component and one adenocarcinoma component (most frequently, invasive breast carcinoma-no special type) [10]. The differential diagnosis of metaplastic carcinoma includes malignant phyllodes tumor, fibromatosis or scar, and, less likely, primary breast sarcoma. A broad panel of CK panels along with P63 is often necessary for diagnosis. In a well-sampled pure spindle cell lesion, unequivocal staining with CK or P63 is deemed sufficient to diagnose metaplastic carcinoma [9].

Treatment modalities for breast carcinosarcoma correspond to other common pathological types of breast malignancy. Practically, mastectomy is proven beneficial and indicated. Some centers provide chemotherapy and radiotherapy as adjuvant therapy but the efficacy is not well established yet in controlling the disease as the reported cases are sparse and recurrence has been reported [11]. Chen et al. reported that systemic chemotherapy generally contributes to poor response toward this type of tumor and requires further investigation [12]. Our patient had a rapid growth from 1-2 cm to a massive breast mass in merely six months, which caused her to suffer pain, anemia, thrombocytosis, coagulopathy, and hypoalbuminemia. Due to her difficult diagnosis and general condition, surgery was unavoidable. If this has been known prior, the best treatment option for her was neoadjuvant treatment, followed by surgery after cytoreduction.

Studies have shown that hormonal therapy is rarely offered to patients with carcinosarcoma as the incidence of positivity toward receptors is low. In our patient, ER, PR, and HER2-neu receptors were negative. Rosen et al. described that the absence of ER and PR most probably elicits the absence of a prominent glandular epithelial compartment in these tumors [7].

This tumor metastasizes lymphogenously and hematogenously. For hematogenous metastasis, pleural and pulmonary spread are more common compared to the brain, liver, or bones [11]. In our patient, there was no evidence of distant metastasis preoperatively. After surgical resection of the tumor, watchful follow-up and surveillance are mandatory as recurrence can be expeditious and devastating. Recommended follow-up visits are three-monthly follow-ups in the initial year and subsequently six-monthly follow-ups.

## Conclusions

Carcinosarcoma of the breast is challenging to diagnose as it is uncommon and fast-growing. Breast imaging and pre-surgery samples may not be diagnostic. Definitive surgery is indicated, while the role of adjuvant treatments such as chemotherapy and radiotherapy is still uncertain due to the small number of reported cases. More case studies and reports regarding appropriate treatment strategies as well as surveillance are required. Careful follow-up of the patient is crucial.
